# A room-temperature magnetic semiconductor from a ferromagnetic metallic glass

**DOI:** 10.1038/ncomms13497

**Published:** 2016-12-08

**Authors:** Wenjian Liu, Hongxia Zhang, Jin-an Shi, Zhongchang Wang, Cheng Song, Xiangrong Wang, Siyuan Lu, Xiangjun Zhou, Lin Gu, Dmitri V. Louzguine-Luzgin, Mingwei Chen, Kefu Yao, Na Chen

**Affiliations:** 1 School of Materials Science and Engineering, Tsinghua University, Beijing 100084, China; 2 Beijing Laboratory for Electron Microscopy, Institute of Physics, CAS, Beijing 100190, China; 3 WPI Advanced Institute for Materials Research (WPI-AIMR), Tohoku University, Sendai 980-8577, Japan; 4 Physics Department, The Hong Kong University of Science and Technology, Clear Water Bay, Kowloon, Hong Kong 999077, China; 5 HKUST Shenzhen Research Institute, Shenzhen 518057, China

## Abstract

Emerging for future spintronic/electronic applications, magnetic semiconductors have stimulated intense interest due to their promises for new functionalities and device concepts. So far, the so-called diluted magnetic semiconductors attract many attentions, yet it remains challenging to increase their Curie temperatures above room temperature, particularly those based on III–V semiconductors. In contrast to the concept of doping magnetic elements into conventional semiconductors to make diluted magnetic semiconductors, here we propose to oxidize originally ferromagnetic metals/alloys to form new species of magnetic semiconductors. We introduce oxygen into a ferromagnetic metallic glass to form a Co_28.6_Fe_12.4_Ta_4.3_B_8.7_O_46_ magnetic semiconductor with a Curie temperature above 600 K. The demonstration of *p*–*n* heterojunctions and electric field control of the room-temperature ferromagnetism in this material reflects its *p*-type semiconducting character, with a mobility of 0.1 cm^2^ V^−1^ s^−1^. Our findings may pave a new way to realize high Curie temperature magnetic semiconductors with unusual multifunctionalities.

## Abstract (web summary)

Magnetic semiconductors provide control of spin states in addition to charge states realized in conventional semiconductors, yet currently limited to weak magnetism at low temperature. Liu *et al*. introduce oxygen into a ferromagnetic metallic glass, resulting in a Curie temperature above 600 K.

Magnetic semiconductors (MSs) hold a very special position in the field of spintronics because they allow effective manipulation of both charge and spin. This feature is important in devices combining logic functionalities and information storage capabilities. The existing technology to obtain diluted MSs (DMSs) is to dope magnetic elements into traditional semiconductors^1^,^2^,^3^,^4^. A successful example is the manganese-doped GaAs system, which shows a relatively high Curie temperature up to 200 K (ref. 5). Although ferromagnetism of DMSs at or above room temperature has been reported in various systems^6^,^7^, the distribution of magnetization is usually heterogeneous and the obtained ferromagnetism is too weak to be comparable to that of the conventional ferromagnetic materials^8^,^9^,^10^,^11^. The lack of MSs with strong magnetism above room temperature becomes, therefore, a bottleneck of the fundamental development of MS-based spintronic/electronic devices.

Amorphous metal oxide semiconductors are of both fundamental and technological interest owing to their high carrier mobility and large-area uniformity^12^,^13^. Following the tremendous demands of transparent materials for flexible electronic devices including e-paper, sensors and optical detectors, amorphous metal oxides are potential candidates that can partially replace the silicon-based semiconductors. These visually transparent semiconductors are, however, usually non-ferromagnetic.

Metallic glasses (MGs) and amorphous metal oxides, both lack of long-range order in atomic arrangements, have distinct properties. The superior mechanical performance coupled with their unique chemical and physical properties distinguishes MGs as attractive materials that can be utilized in micro-/nano-electro- mechanical system devices, where the integration of optical, electrical and magnetic components is required^14^,^15^,^16^,^17^,^18^. In fact, MG thin films have proven to be advanced metal–insulator–metal electrode materials by holding very smooth surfaces with a root mean squared roughness down to ∼0.2 nm (ref. 19).

Here we propose to introduce non-magnetic oxygen into originally ferromagnetic metal-based MGs to convert them into MSs. On the basis of this idea, we fabricate an amorphous MS by utilizing a Co-Fe-Ta-B system, and realize a unique combination of optical, electrical and ferromagnetic properties in one single material. Such an unusual MS is produced by a magnetron sputter deposition method, which has already been used extensively in semiconductor industry to deposit thin films of various materials in integrated circuit processing.

## Results

### A room-temperature MS from a ferromagnetic MG

**Figure 1:**
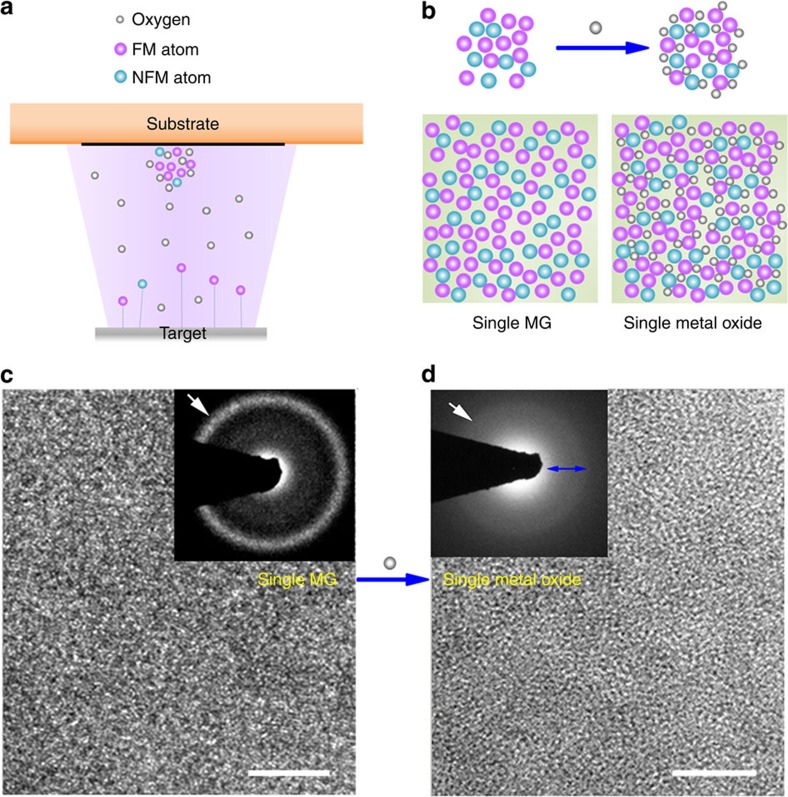
Formation of an amorphous metal oxide from a ferromagnetic metallic glass. (**a**) Schematic diagram illustrating deposition of thin films. (**b**) Oxidation mechanism of the ferromagnetic (FM)–non-ferromagnetic (NFM) system. (**c**,**d**) High-resolution transmission electron microscopy images of the single-phase Co_44_Fe_20_Ta_10_B_26_ MG ribbon and the single-phase Co_28.6_Fe_12.4_Ta_4.3_B_8.7_O_46_ amorphous metal oxide. The insets are the corresponding selected-area electron diffraction patterns of the MG ribbon and the amorphous metal oxide, respectively. Scale bars, 5 nm.

The introduction of oxygen to a Co-Fe-Ta-B system is performed by magnetron sputtering under a gas mixture of argon and oxygen (Fig. 1a). In this way, a single metal oxide Co_28.6_Fe_12.4_Ta_4.3_B_8.7_O_46_ (in atomic percentage) is produced from a ferromagnetic MG precursor (Fig. 1b).

The Co_28.6_Fe_12.4_Ta_4.3_B_8.7_O_46_ thin film exhibits an amorphous structure with a maze-like pattern, similar to that of the Co_44_Fe_20_Ta_10_B_26_ MG ribbon (Fig. 1c,d). Nevertheless, the corresponding selected-area electron diffraction patterns reveal their local structural difference. The amorphous Co_28.6_Fe_12.4_Ta_4.3_B_8.7_O_46_ (a-CFTBO) thin film presents one halo adjacent to the direct beam spot, which is much broader than that of the MG ribbon (insets of Fig. 1c,d). This indicates a more homogeneously disordered structure formed in the metal oxide phase.

**Figure 2:**
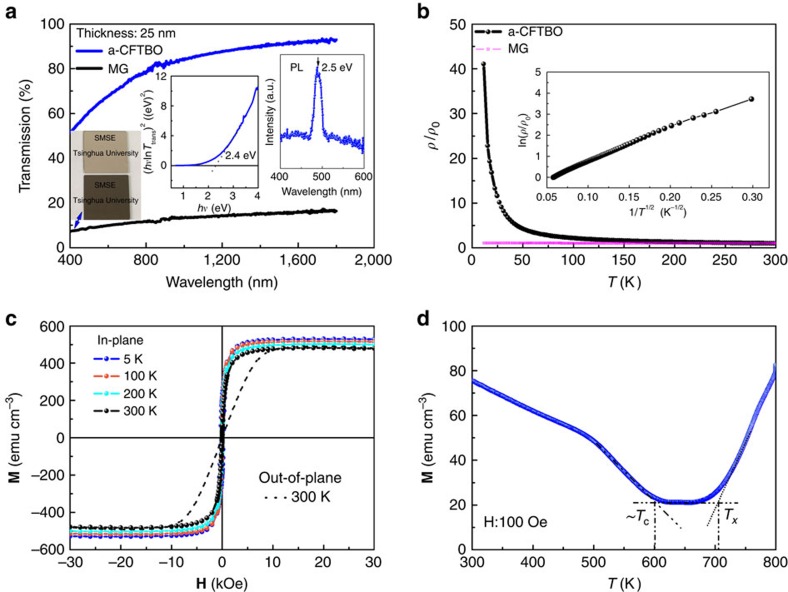
Properties of the a-CFTBO thin films. (**a**) The optical transmission spectra of the a-CFTBO and the MG thin films at the same thickness of 25 nm. Left inset shows the Tauc plot derived from the transmission spectrum of the a-CFTBO thin film. Right inset shows its room-temperature photoluminescence (PL) spectrum. (**b**) The normalized resistivity *ρ*/*ρ*_0_ as a function of temperature (*ρ*_0_ is the room temperature resistivity). Inset is the plot of ln(*ρ*/*ρ*_0_) versus 1/*T*^1/2^ based on the experimental data. (**c**) Magnetic field dependence of the magnetization (**M**–**H** curves) measured at different temperatures. (**d**) Temperature dependence of the magnetization measured under an in-plane magnetic field of 100 Oe.

Inclusion of oxygen in the MG precursor opens its optical bandgap, which is estimated to be about 2.4 eV from the Tauc plot based on an optical transmission spectrum of the a-CFTBO thin film (inset of Fig. 2a). As a result, the a-CFTBO thin film, which has a thickness of 25 nm, is much more transparent than the MG thin film at the same thickness (Fig. 2a). Owing to photoelectric interaction in the material, a characteristic photoluminescence spectrum at room temperature is observed at a wavelength of 490 nm, corresponding to a direct bandgap of about 2.5 eV (inset of Fig. 2a). This is consistent well with its optical bandgap.

The a-CFTBO thin film shows a negative temperature dependence of resistivity, indicative of a non-metallic behaviour (Fig. 2b). In comparison, the resistivity of the MG ribbon changes linearly with temperature by yielding an extremely small temperature coefficient of order of 10^−5^ K^−1^, in agreement with electrical behaviour of conventional MGs (Fig. 2b). In addition, the room-temperature resistivity of the a-CFTBO thin film is of order of 1 Ω cm (in the range of 10^−3^ to 10^12^ Ω cm for semiconductors), which is higher than that of the MG ribbon (∼10^−4^ Ω cm) by about four orders of magnitude. Moreover, its resistivity shows a negative temperature dependence of ln(*ρ*/*ρ*_0_)∝1/*T*^1/2^ (inset of Fig. 2b), characteristic of a semiconductor^20^.

In addition to its semiconducting properties, the a-CFTBO thin film sustains its intrinsic ferromagnetic properties. Owing to the shape anisotropy, the a-CFTBO thin film with a thickness of 100 nm has an in-plane axis of easy magnetization, as expected (Fig. 2c). The temperature dependence of its magnetization gives a Curie temperature higher than 600 K (Fig. 2d). A residual magnetization of ∼20 emu cm^−3^ can be observed in the temperature range from 600 to 705 K. This originates from structural relaxation and nucleation of crystals before apparent crystal growth occurring in the a-CFTBO thin film. At about 705 K the magnetization starts to increase due to such crystallization, consistent with early findings that nanocrystallized CoFeMB (M=Ta, Hf) thin film has a higher Curie temperature than the corresponding amorphous CoFeMB smaple^21^,^22^. The ferromagnetism together with its semiconducting properties indicates that the single-phase a-CFTBO thin film is a MS.

### Magnetotransport properties of the a-CFTBO MS

**Figure 3:**
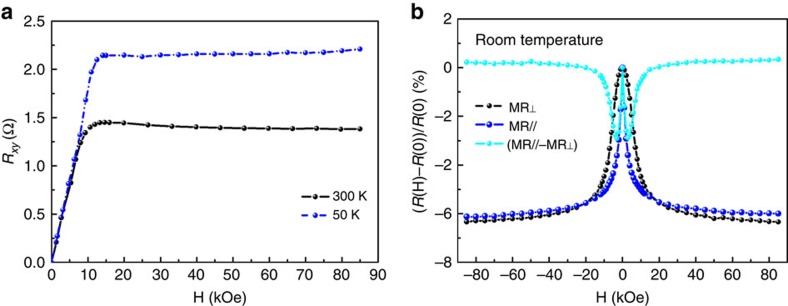
Magnetotransport properties of the *p*-type a-CFTBO MS. (**a**) Magnetic field dependence of the Hall resistance (*R*_*xy*_) measured at 1 mA, mainly resulting from the anomalous Hall effect. (**b**) Magnetic field dependence of the anisotropic magnetoresistance by measuring the changes of the electrical resistance in an applied field either parallel or perpendicular to the current, which are indicated as MR// or MR_⊥_.

The Hall resistance *R*_*xy*_ can be expressed as


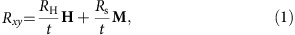


where *R*_H_ is the ordinary Hall coefficient, *R*_s_ is the anomalous Hall coefficient, *t* is the thickness of the thin film, **H** is the applied magnetic field and **M** is the magnetization of the sample. An anomalous Hall effect is obtained (Fig. 3a), which occurs typically in a ferromagnetic solid as a consequence of spin–orbit coupling^23^. The slope of the ordinary Hall effect in the *R*_*xy*_-**H** curve measured at 50 K under magnetic fields up to 85 kOe reveals that the electric conduction is *p*-type with a hole concentration of order of 10^20^ cm^−3^ (Fig. 3a). The mobility is ∼0.1 cm^2^ V^−1^ s^−1^. Structurally, the composition of the a-CFTBO thin film can be written in form of (FM_*x*_NFM_1−*x*_)O_*n*−*δ*_, where FM denotes ferromagnetic metals, NFM denotes non-ferromagnetic elements, *n* denotes a fully compensated state and *δ* denotes oxygen deficiency. In this sense, a huge number of holes are supposed to arise from the oxygen deficiency-induced oxygen vacancies in the amorphous metal oxide, which therefore account for its *p*-type conductivity.

The anisotropic magnetoresistance is observed in the a-CFTBO MS by measuring the changes of its electrical resistance in an applied field either parallel or perpendicular to the current, which are indicated as MR// or MR_⊥_ (Fig. 3b). At low fields in which the saturation magnetization is being approached, both MR// and MR_⊥_ decrease significantly with the field (Fig. 3b). At high fields, they reach a saturation value of around −6% as the magnetization of the thin film saturates. These curves are in line with the in-plane and out-of-plane magnetization of the a-CFTBO MS (Fig. 2c). In addition, the anisotropic magnetoresistance effect of the a-CFTBO MS is also similar to that observed in the single-phase (Ga,Mn)As DMS^24^,^25^.

### A *p*–*n* heterojunction based on the *p*-type a-CFTBO MS

**Figure 4:**
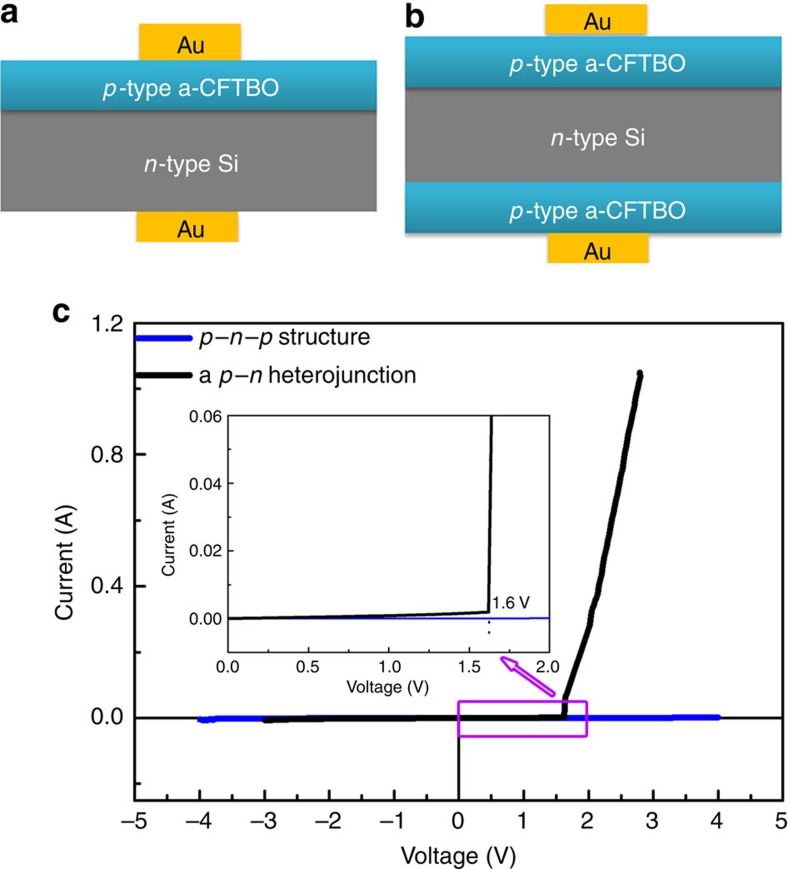
Fabrication of *p*–*n* heterojunctions composed of the *p*-type a-CFTBO MS. (**a**) Schematic of *p*–*n* heterojunction structure of Au (10 nm)/*p*-type a-CFTBO (300 nm)/*n*-type Si (300 μm)/Au (10 nm). (**b**) Schematic of *p*–*n*–*p* structure of Au (10 nm)/*p*-type a-CFTBO (300 nm)/*n*-type Si (300 μm)/*p*-type a-CFTBO (300 nm)/Au (10 nm). The *n*-type single-crystal Si used in the heterojunctions is heavily doped with phosphorous and has a resistivity of order of 10^−3^ Ω cm. (**c**) Current–voltage curves of the *p*–*n* heterojunctions. The inset gives an enlargement of the part indicated by a rectangle in the current–voltage curves.

Since most of the amorphous metal oxide semiconductors are *n*-type and are notoriously hard to be doped into *p*-type, formation of the *p*-type MS in this study would help to realize *p*–*n* junctions based on amorphous oxide semiconductors, which are the origin of various active functions of semiconductors^26^. We hence fabricated a rudimentary *p*–*n* heterojunction with a structure of Au/*p*-type a-CFTBO/*n*-type Si/Au as shown in Fig. 4a. To further validate that the *p*–*n* junction is able to allow electrical current to flow in one direction, yet not in the opposite direction, the *p*–*n*–*p* structure was also fabricated as shown in Fig. 4b. The current–voltage curve of the *p*–*n* junction exhibits a rectifying characteristic with a threshold voltage of 1.6 V (inset of Fig. 4c). In addition, no current flows through the *p*–*n*–*p* structure as expected (Fig. 4c). These experimental results consolidate that the *p*-type a-CFTBO MS can fulfil the basic functionalities of the heterojunctions and also demonstrate its potential for device applications.

## Discussion

Although the classification of metals, insulators and semiconductors refers to crystals with periodic structures in the classical band theory, such definition can also be extended to amorphous materials. Insulators and semiconductors are those whose Fermi levels lie in the energy gaps between two nearby mobility edges (boundaries between localized and extended states), while metals are those whose Fermi level lies in the extended states. The term semiconductor in our case is adopted for amorphous semiconductors. Thus, MSs discussed here are those amorphous semiconductors with spontaneous magnetization.

Generally, MSs refer to single-phase crystalline materials in which ferromagnetism and semiconducting properties coexist^27^. Such definition of crystalline MSs is obviously inapplicable to amorphous semiconductors, which lack periodic lattice structures. In analogy to those crystalline MSs, amorphous MSs refer to non-crystalline solids in which, ferromagnetism and semiconducting properties coexist.

For crystalline MSs, it is essential to have an exchange interaction between the carriers in the semiconducting band and the localized electrons of the magnetic ions occupying the lattice of the host semiconductors^10^. As already demonstrated in III–V DMSs, electric field control of ferromagnetism can be realized due to this interaction^1^,^4^. Nevertheless, the Curie temperature of the III–V DMSs is lower than room temperature, which poses a significant hurdle to broad applications. For amorphous MSs, the charge carriers contributing to their semiconducting properties are therefore expected to show exchange interaction with the randomly distributed magnetic moments as well.

**Figure 5:**
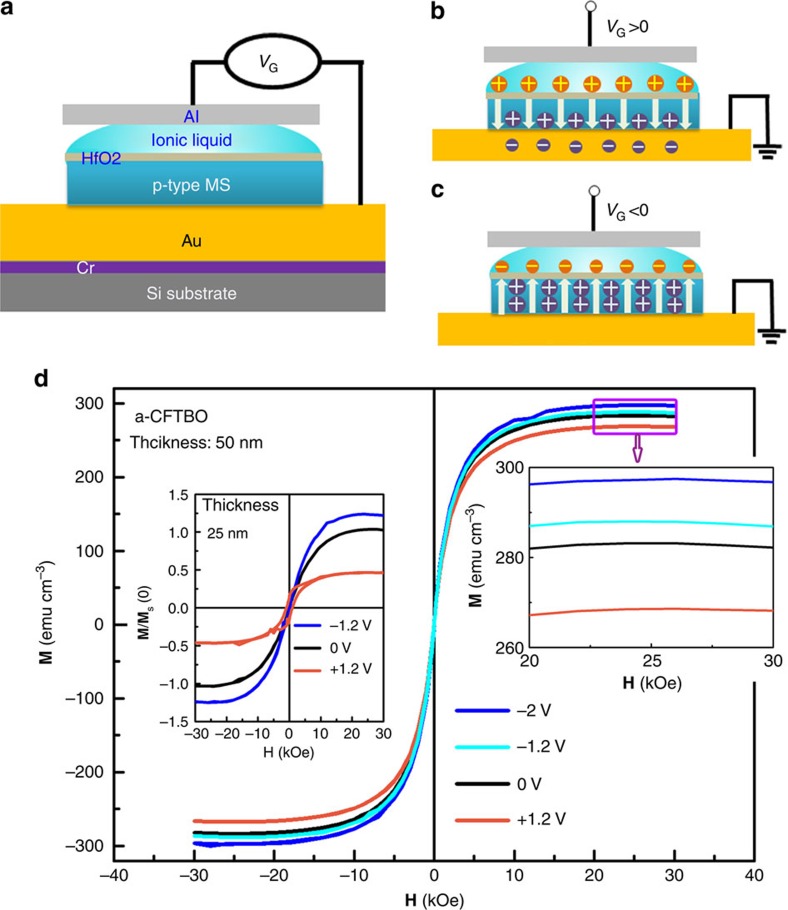
Electric field control of ferromagnetism in the a-CFTBO MS. (**a**) Schematic of the experimental set-up for applying gate voltage on the a-CFTBO thin film through a drop of ionic liquid. The thickness of an insulating HfO_2_ layer is about 2 nm. (**b**) Illustration of electrically induced decrease of carrier concentration at a positive gate voltage. (**c**) Illustration of electrically induced increase of carrier concentration at a negative gate voltage. (**d**) Variation of **M**–**H** curves with different gate voltages measured at 300 K. The thickness of the a-CFTBO MS is 50 nm. Left inset shows the plot of the normalized **M**/**M**_s_(0) versus magnetic field measured at different gate voltages for the a-CFTBO MS with a thickness of 25 nm, **M**_s_(0) denotes the saturation magnetization without any gate voltage. Right inset is an enlargement of the part indicated by a rectangle.

To probe such exchange interaction between the carriers and the local magnetic moments in the a-CFTBO MS, the electric field-induced change in its saturation magnetization (**M**_s_) was measured by altering the gate voltage (*V*_G_) exerted by an ionic liquid as shown in Fig. 5a. Such method has proven to be effective for realizing stable modulation of carrier densities even when the gate voltage is removed^28^,^29^,^30^. When *V*_G_ is positive, the holes move along the direction of the electric field and some of them annihiliate once combined with electrons from the Au electrode (Fig. 5b). As a result, the carrier concentration of the a-CFTBO MS decreases, leading to a reduction of its **M**_s_. In contrast, a negative *V*_G_ enables the carrier concentration to increase, thus enhancing its **M**_s_ (Fig. 5c). By varying *V*_G_ on the a-CFTBO MS with a thickness of 50 nm from −2 to 1.5 V, the measured **M**_s_ decreases from 297 to 268 emu cm^−3^ at 300 K (Fig. 5d). As the thickness of the a-CFTBO MS is reduced from 50 to 25 nm, the affected zone by the ionic liquid gate increases. Consequently, the change in **M**_s_ becomes more significant (inset of Fig. 5d). These experimental results indicate that the magnetic behaviour of the a-CFTBO MS can be modulated by electrical means, which permits an external control of the room-temperature ferromagnetism. Moreover, our observations also demonstrate the presence of exchange interaction between the carriers and the localized magnetic moments in the a-CFTBO MS, which is the key to utilize the interplay of semiconductivity with ferromagnetism.

For MGs, oxygen is usually a harmful impurity element because of its detrimental effects on both glass-forming ability and mechanical properties, mostly associated with crystalline oxide precipitates^31^. However, the introduction of oxygen to the present material is favoured, mediating the transition from a MG conductor to an amorphous semiconductor. Such a fascinating emergence of combined functionalities (optical, electrical and ferromagnetic) in one novel material has not yet been reported before, which provides a fertile ground for creating new physics and device concepts. The approach of introducing light atoms such as oxygen into a ferromagnetic amorphous metal opens up an avenue to make high Curie temperature MSs that may allow MS-based spintronic/electronic devices to be operable at room temperature. Such a method may be further extended to the crystalline ferromagnetic metals for producing new materials with unique physical properties.

## Methods

### Sample preparation and structural characterization

The ferromagnetic Co-Fe-Ta-B-O thin films with thickness of <400 nm were deposited on Si and quartz glass substrates by using magnetron sputtering with an alloy target under a gas mixture of argon and oxygen^32^,^33^. The average composition was determined by using an electron probe micro-analyser equipment. The structure of the thin films was investigated using high-resolution transmission electron microscopy.

### Property measurements

The optical transparency of the samples was measured by using Jasco V-650 UV-vis Spectrophotometer (Lambda 950 UV/Vis/NIR Spectrophotometer). The photoluminescence spectrum of the samples was measured by using RM1000 Raman Microscope. Electrical properties of the samples were measured using Physical Property Measurement System (PPMS-9, Quantum Design). The gate voltage was applied using an Agilent 2901 A instrument. All of the magnetic measurements were conducted after maintaining *V*_G_ for 30 min without special instruction. Magnetic properties, for instance, magnetic moment–temperature relations, were measured using SQUID-VSM (Quantum Design).

### Data availability

The data that support the findings of this study are available from the corresponding author on reasonable request.

## Additional information

**How to cite this article**: Liu, W. *et al*. A room-temperature magnetic semiconductor from a ferromagnetic metallic glass. *Nat. Commun.* **7**, 13497 doi: 10.1038/ncomms13497 (2016).

**Publisher's note:** Springer Nature remains neutral with regard to jurisdictional claims in published maps and institutional affiliations.
